# Protected area downgrading, downsizing, and degazettement as a threat to iconic protected areas

**DOI:** 10.1111/cobi.13365

**Published:** 2019-07-17

**Authors:** Siyu Qin, Rachel E. Golden Kroner, Carly Cook, Anteneh T. Tesfaw, Rowan Braybrook, Carlos Manuel Rodriguez, Claire Poelking, Michael B. Mascia

**Affiliations:** ^1^ Moore Center for Science Conservation International 2011 Crystal Drive Arlington VA 22202 U.S.A.; ^2^ Department of Environmental Science and Policy George Mason University 4400 University Drive Fairfax VA 22030 U.S.A.; ^3^ School of Biological Sciences Monash University 25 Rainforest Walk Clayton VIC 3800 Australia; ^4^ Policy Center for Environment and Peace Conservation International 2011 Crystal Drive Arlington VA 22202 U.S.A.

**Keywords:** biodiversity conservation, governance, PADDD, UNESCO, World Heritage Sites, conservación de la biodiversidad, gobernanza, PADDD, Sitios de Patrimonio Mundial, UNESCO, 生物多样性保护, 管治, PADDD, UNESCO, 世界遗产地

## Abstract

Protected areas (PAs) are expected to conserve nature and provide ecosystem services in perpetuity, yet widespread protected area downgrading, downsizing, and degazettement (PADDD) may compromise these objectives. Even iconic protected areas are vulnerable to PADDD, although these PADDD events are often unrecognized. We identified 23 enacted and proposed PADDD events within World Natural Heritage Sites and examined the history, context, and consequences of PADDD events in 4 iconic PAs (Yosemite National Park, Arabian Oryx Sanctuary, Yasuní National Park, and Virunga National Park). Based on insights from published research and international workshops, these 4 cases revealed the diverse pressures brought on by competing interests to develop or exploit natural landscapes and the variety of mechanisms that enables PADDD. Knowledge gaps exist in understanding of the conditions through which development pressures translate to PADDD events and their impacts, partially due to a lack of comprehensive PADDD records. Future research priorities should include comprehensive regional and country‐level profiles and analysis of risks, impacts, and contextual factors related to PADDD. Policy options to better govern PADDD include improving tracking and reporting of PADDD events, establishing transparent PADDD policy processes, coordinating among legal frameworks, and mitigating negative impacts of PADDD. To support PADDD research and policy reforms, enhanced human and financial capacities are needed to train local researchers and to host publicly accessible data. As the conservation community considers the achievements of Aichi Target 11 and moves toward new biodiversity targets beyond 2020, researchers, practitioners, and policy makers need to work together to better track, assess, and govern PADDD globally.

## Abstract 2

*Article impact statement*: Legal changes affect even iconic protected areas. Sustaining conservation progress requires research, policies, and human‐capacity investments.

## Abstract (es)

Cambios de Categoría, Reducción del Tamaño y Eliminación de las Listas de Protección como Amenazas para las Áreas Protegidas Icónicas

**Resumen**

Se espera que las áreas protegidas (PAs, en inglés) conserven la naturaleza y proporcionen servicios ambientales a perpetuidad, sin embargo las extensas prácticas de reducción del tamaño, eliminación de las listas de protección y cambios de categoría de las áreas protegidas (PADDD, en inglés) pueden poner en riesgo a estos objetivos. Incluso las áreas protegidas icónicas son vulnerables a los PADDD, aunque estos eventos de PADDD comúnmente no se reconocen. Identificamos 23 eventos de PADDDD promulgados y propuestos dentro de sitios de Patrimonio Natural Mundial y examinamos la historia, el contexto y las consecuencias de los eventos PADDD en cuatro PAs icónicas (el Parque Nacional Yosemite, el Santuario del Oryx Árabe, el Parque Nacional Yasuní y el Parque Nacional Virunga). Con base en el conocimiento obtenido de investigaciones publicadas y talleres internacionales, estos cuatro casos revelaron las diferentes presiones que traen consigo los intereses en competencia por desarrollar o explotar los paisajes naturales y la variedad de mecanismos que faciliten las PADDD. Existen vacíos de conocimiento en el entendimiento de las condiciones a través de las cuales las presiones del desarrollo se transforman en eventos PADDD y los impactos que tienen, parcialmente debido a la falta de registros completos de los eventos PADDD. Las prioridades de las próximas investigaciones deberían incluir perfiles completos a nivel regional y nacional y un análisis de riesgo, impactos y factores contextuales relacionados con los PADDD. Las opciones políticas para gobernar de mejor manera los PADDD incluyen la mejora del rastreo y del reporte de eventos PADDD, el establecimiento de procesos políticos transparentes para los PADDD, la coordinación entre los marcos de trabajo legales y la mitigación de los impactos negativos de los PADDD. Para apoyar la investigación de los PADDD y las reformas políticas se requiere de una mayor capacidad humana y financiera para entrenar a los investigadores locales y para acoger datos accesibles para el público. Conforme la comunidad de la conservación considera los logros del Objetivo 11 de Aichi y se posiciona hacia nuevos objetivos para la biodiversidad más allá del 2020, los investigadores, los practicantes y los legisladores necesitan trabajar en conjunto para rastrear, evaluar y gobernar de mejor manera los PADDD a nivel mundial.

## 摘要

建立保护区 (PA) 的初衷是永久性地保护自然和提供生态系统服务, 然而广泛的保护区降级、缩减和撤销 (PADDD) 或将危及这些目标。即便是标志性的保护区也未必免于 PADDD, 尽管这些PADDD未被注意。我们确认了发生于世界自然遗产地中的 23 起 PADDD 事件, 并分析了四个标志性保护区 (优胜美地国家公园、阿拉伯羚羊保护区、亚苏尼国家公园和维龙加国家公园) 中的 PADDD 事件及其历史、背景和后果。基于已发表的研究和研讨会中的见解, 这 4 个案例进一步揭示了各种开发利用自然的压力、不同利益间的讨价还价、和实现 PADDD 的多种机制。对于在何种情况下这些开发压力会转换为 PADDD 事件并导致何种影响, 我们的理解还存在缺口, 部分原因是缺乏相对完整的 PADDD 记录。未来的研究重点应当包括建立完整的区域及国家的 PADDD 概况, 分析 PADDD 的风险、影响和相关的情景因素。更好管治 PADDD 的政策则包括优化对 PADDD 事件的记录和报告, 建立透明的 PADDD 政策流程, 协调法律框架以及减缓PADDD的负面影响。为支持 PADDD 研究和政策改进, 尚需增加人力及财力投入以培训当地研究人员和存放公众可获取的数据。随着保护界评估爱知目标 11 的成就并向 2020 年后的新生物多样性目标迈进, 研究人员、保护从业者及政策制定者需要携手合作, 以更好地在全球层面上记录、评估、和管理 PADDD。

## Introduction

National parks and other protected areas (PAs) are key components of the conservation toolbox. The global PA estate has grown from a handful of sites in 1900 to over 200,000 PAs today, covering approximately 14.9% of terrestrial areas and inland waters and 7.3% of marine and coastal areas (UNEP‐WCMC, IUCN, & NGS 2018). Throughout the modern history of PAs, their creation has been motivated by the need to protect spectacular landscapes, conserve biodiversity, and support ecosystems services (Watson et al. 2014). However, PA effectiveness varies by geography, PA governance and other characteristics, socioecological context, and management capacity (Joppa & Pfaff 2010; Pfaff et al. 2014; Gill et al. 2017).

Despite the net growth in protected lands and waters, research (Mascia et al. 2014; Forrest et al. 2015; Pack et al. 2016; Cook et al. 2017; Golden Kroner et al. 2019) reveals widespread, albeit underreported, protected area downgrading, downsizing, and degazettement (PADDD). Downgrading is a decrease in legal restrictions on the number, magnitude, or extent of human activities within a PA; downsizing is a decrease in the size of a PA as a result of excision of an area of land or sea area through a legal boundary change; and degazettement is a loss of legal protection for an entire PA (Mascia & Pailler 2011). More than 3,700 enacted PADDD events have been documented in 73 countries from 1892 to 2018, affecting about 2 million km^2^ (Golden Kroner et al. 2019). Proximate causes of PADDD include industrial‐scale resource extraction and development, local land pressures and land claims, and, to a much lesser extent, conservation planning (Mascia et al. 2014). Although the Convention on Biological Diversity (CBD) calls for protecting 17% of terrestrial area by 2020, PADDD not only hinders national progress toward Aichi Target 11 (Mascia et al. 2014), but may also accelerate tropical deforestation and carbon emissions (Forrest et al. 2015) and exacerbate habitat fragmentation (Golden Kroner et al. 2016).

Emerging evidence indicates that even iconic PAs are vulnerable to PADDD (Table 1), including those recognized by United Nations Educational, Scientific, and Cultural Organization (UNESCO) for their outstanding values (Mascia et al. 2014; Allan et al. 2017). More than a quarter of UNESCO World Heritage Sites are threatened by existing or proposed oil and gas extraction (Osti et al. 2011; Veillon 2014), an activity incompatible with World Heritage status (UNESCO 2017a). The underlying drivers of PADDD often differ by country and context due to differences in legal frameworks, socioeconomic contexts, and political dynamics, making it challenging to generalize about drivers and impacts.

**Table 1:** Enacted and proposed PADDD^a^ events in UNESCO World Heritage Sites.^b^

Country	UNESCO World Heritage Site	Year decision enacted (proposed)	PADDD type	Proximate cause	Year decision reversed
Brazil	Iguaçu National Park	(1998)	downsize	infrastructure	2004
	Iguaçu National Park	(2010)	downgrade	infrastructure	
Bulgaria	Pirin National Park	2012	downgrade	industrialization	2012
DRC	Virunga National Park	2010	downgrade	oil and gas	2014
	Virunga National Park	2015	downgrade	oil and gas	
	Virunga National Park	(2018)	downgrade	oil and gas	
	Salonga National Park	(2018)	downgrade	oil and gas	
Ecuador	Sangay National Park	2004	downsize	multiple causes	
Guinea	Mount Nimba National Park	1993	downsize	mining	
Oman	Arabian Oryx Sanctuary	2007	downsize	oil and gas	
Tanzania	Selous Game Reserve	2012	downsize	mining	
	Selous Game Reserve	2018	downgrade	infrastructure	
	Serengeti National Park	(2010)	downgrade	infrastructure	2011
	Serengeti National Park	(2012)	downsize	infrastructure	2012
U.S.A.	Yellowstone National Park	(2014)	downgrade	other (recreation)	2016
	Everglades National Park	(2011)	downgrade	infrastructure	2012
	Olympic National Park	(2011)	downgrade	infrastructure	2013
	Olympic National Park	(2015)	downgrade	infrastructure	2017
	Yosemite National Park	1892^c^	downgrade	infrastructure	
	Yosemite National Park	1901^c^	downgrade	infrastructure	
	Yosemite National Park	1905^c^	downsize	forestry	
	Yosemite National Park	1906^c^	downsize	forestry	
	Yosemite National Park	1913^c^	downgrade	infrastructure	

a Protected area downgrading, downsizing, and degazettement.

b As documented in http://PADDDtracker.org (CI & WWF 2019) and in this paper.

c The PADDD events enacted before UNESCO designation.

To illustrate the complexity of contexts and mechanisms of PADDD events and stimulate discussion about potential mechanisms to address them, we examined the processes associated with PADDD events in 4 iconic PAs around the world: Yosemite National Park (United States), Arabian Oryx Sanctuary (Oman), Yasuní National Park (Ecuador), and Virunga National Park (Democratic Republic of Congo). In each case we considered the context, timing, area affected, enabling mechanisms, and proximate causes of PADDD, impacts of PADDD, and the potential future of each PA. In addition to case‐specific lessons learned, we drew on evidence from a range of sources to highlight opportunities for policy reform and areas for capacity building that are needed to improve the transparency of PADDD governance. These lessons also reveal priorities for research to advance scientific understanding of PADDD and develop strategies to address this emerging issue.

## PADDD Case Studies

### Yosemite National Park

First protected as a land grant in 1864 and then as a national park in 1890, Yosemite National Park is one of the oldest and most popular national parks in the world. The park became a World Heritage Site in 1984 for both its geological and ecological values (UNESCO 1984) and attracts over 4 million visitors annually (USNPS 2018).

**Figure 1:**
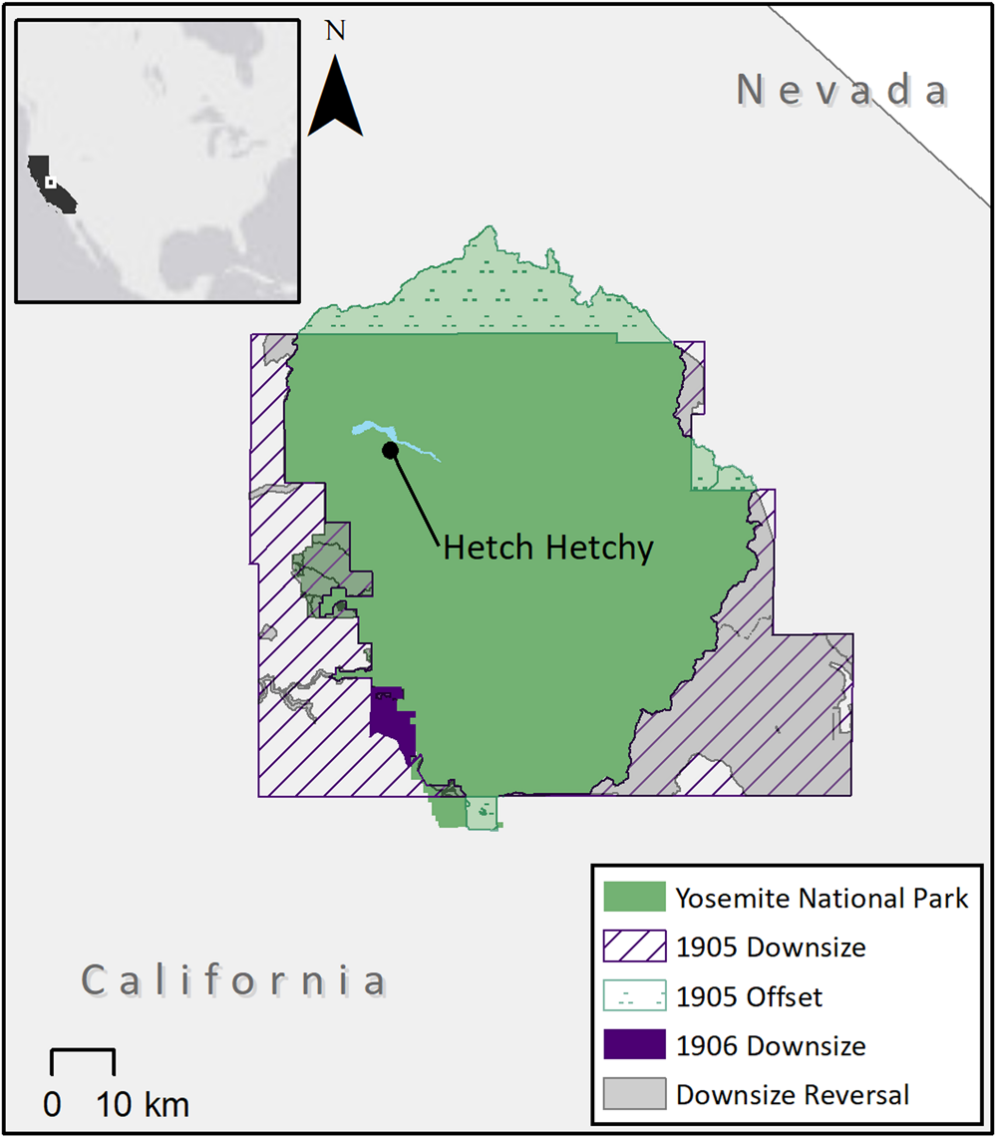
Changes in Yosemite National Park (U.S.A.) land area from 1892 to 2018. The park was repeatedly downgraded (1892, 1901, 1913) to allow construction of roads, facilities, and dams and repeatedly downsized (1905, 1906) for forestry and mining activities. The downsize in 1905 was partially mitigated by a spatial offset and partially reversed in 1964. The park was expanded in 1905, 1914, 1930, 1932, 1937, and 2016. From an original size of 3,886 km^2^, Yosemite now covers ∼2,995 km^2^. Another 1,222 km^2^ are protected (but managed separately) as wilderness areas.

Legal changes to Yosemite's regulations and boundaries occurred early in its history (Fig. 1). The park was downgraded in 1892 to allow construction of wagon roads and turnpikes; in 1901 for electrical lines, dams, and pipes; and in 1913 for the construction of O'Shaughnessy Dam in the Hetch Hetchy Valley (Golden Kroner et al. 2016). The park was downsized in 1905 and 1906 to accommodate forestry and mining activities, removing legal protections from 1,309.30 km^2^ (34% of its original 3,886 km^2^) (Golden Kroner et al. 2016). The 1905 downsizing was partially offset through the addition of 293 km^2^ of land to the park (Golden Kroner et al. 2016).

Several reversals and spatial offsets have partially mitigated the effects of these downsizing and downgrading events. With the passage of the Wilderness Act (1964), more than half (57%) of the downsized lands were established as separate wilderness areas in 1964. At least 6 parcels of land have been added to Yosemite National Park (Fig. 1). Today, Yosemite National Park is 77% of its original size; 19% of the originally protected lands are now under other forms of protection (Golden Kroner et al. 2016).

The legacy of the dynamic history of Yosemite National Park is visible on the landscape today. Forests excised from the original Park that remain unprotected today are more fragmented by roads than lands within the park or the adjoining wilderness areas (Golden Kroner et al. 2016). Conversely, lands that regained protection, even decades later, are less fragmented than lands that remain unprotected, demonstrating the value of long‐term land protection and the value of reversing PADDD to maintain ecosystem connectivity (Golden Kroner et al. 2016).

### Arabian Oryx Sanctuary

**Figure 2:**
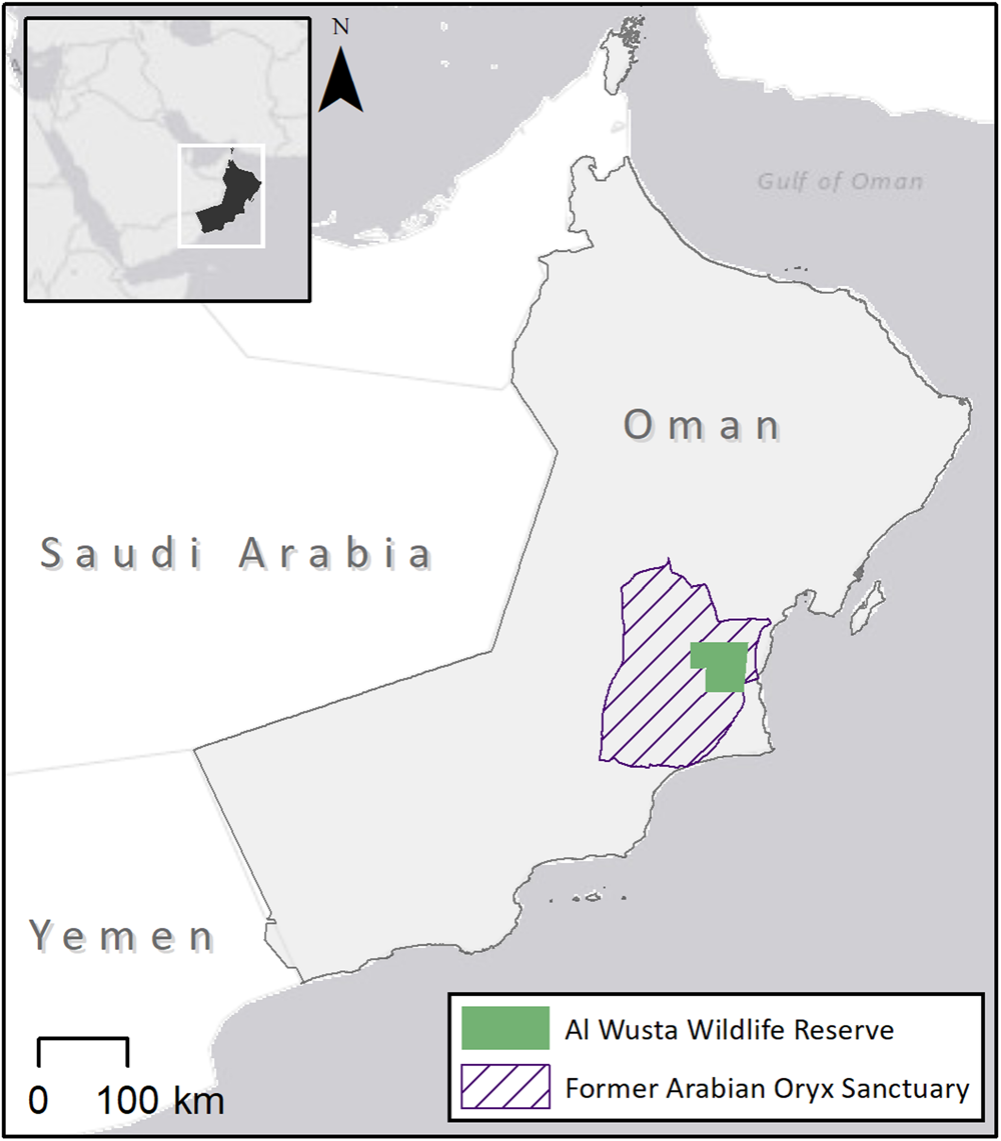
Changes in Arabian Oryx Sanctuary (Oman) boundaries from 1994 to 2016. The Arabian Oryx Sanctuary was downsized in 2007 for hydrocarbon activities and poaching control. From the original size of 34,000 km^2^, the Arabian Oryx Sanctuary (renamed Al Wusta Wildlife Reserve in 2011) now covers 2,824 km^2^.

Established in 1994, the Arabian Oryx Sanctuary (Fig. 2) originally covered 34,000 km^2^ of the central desert and coastal hills of Oman (Government of Oman 1994; Al Jahdhami et al. 2011). In the same year, UNESCO designated 27,500 km^2^ of the sanctuary as a World Heritage Site (UNESCO 1994a). The Sanctuary was best known for the free‐ranging Arabian oryx (*Oryx leucoryx*) population, which was reintroduced to the region in 1982 following the species’ extinction in the wild a decade earlier (Al Jahdhami et al. 2011), and its large Arabian gazelle (*Gazella arabica*) population (IUCN 2017).

Reintroduction of Arabian oryx to the Sanctuary succeeded until the mid‐1990s, when poaching severely decreased the wild population (Al Jahdhami et al. 2011). Citing the impacts of poaching on the oryx population as a major concern, the Omani government downsized the Arabian Oryx Sanctuary by 90% (Government of Oman 2007), a decision opposed by the World Heritage Committee (UNESCO 2007). The remaining 2,824 km^2^ was renamed as the Al Wusta Wildlife Reserve. Because the downsized area coincided with hydrocarbon concession blocks and the decision did not adequately consider ecological impacts, in 2007 UNESCO rescinded the reserve's designation as a World Heritage Site (UNESCO 2007).

Hydrocarbon activities began in the downsized area after the boundary change (Osti et al. 2011). The impact of the downsizing on the wild population of Arabian oryx is unclear because the population had already been depleted by poaching. The Al Wusta Wildlife Reserve was later fenced to prevent poaching, which may restrict the migration of the wild Arabian oryx population and access to water sources during drought (Al Jahdhami et al. 2011). Populations of Arabian oryx and Arabian gazelle have further declined in the reserve, likely due to poaching and ranching within its boundaries (Al Jahdhami et al. 2017). The future of this PA and the species that it protects remain uncertain.

### Yasuní National Park

Established in 1979, Yasuní National Park (Ecuador) is one of the most biodiverse places on the planet (Bass et al. 2010) and home to several indigenous tribes (Finer et al. 2008). Given the global importance of the park for ecological and cultural preservation, UNESCO recognized it as a Biosphere Reserve in 1989.

**Figure 3:**
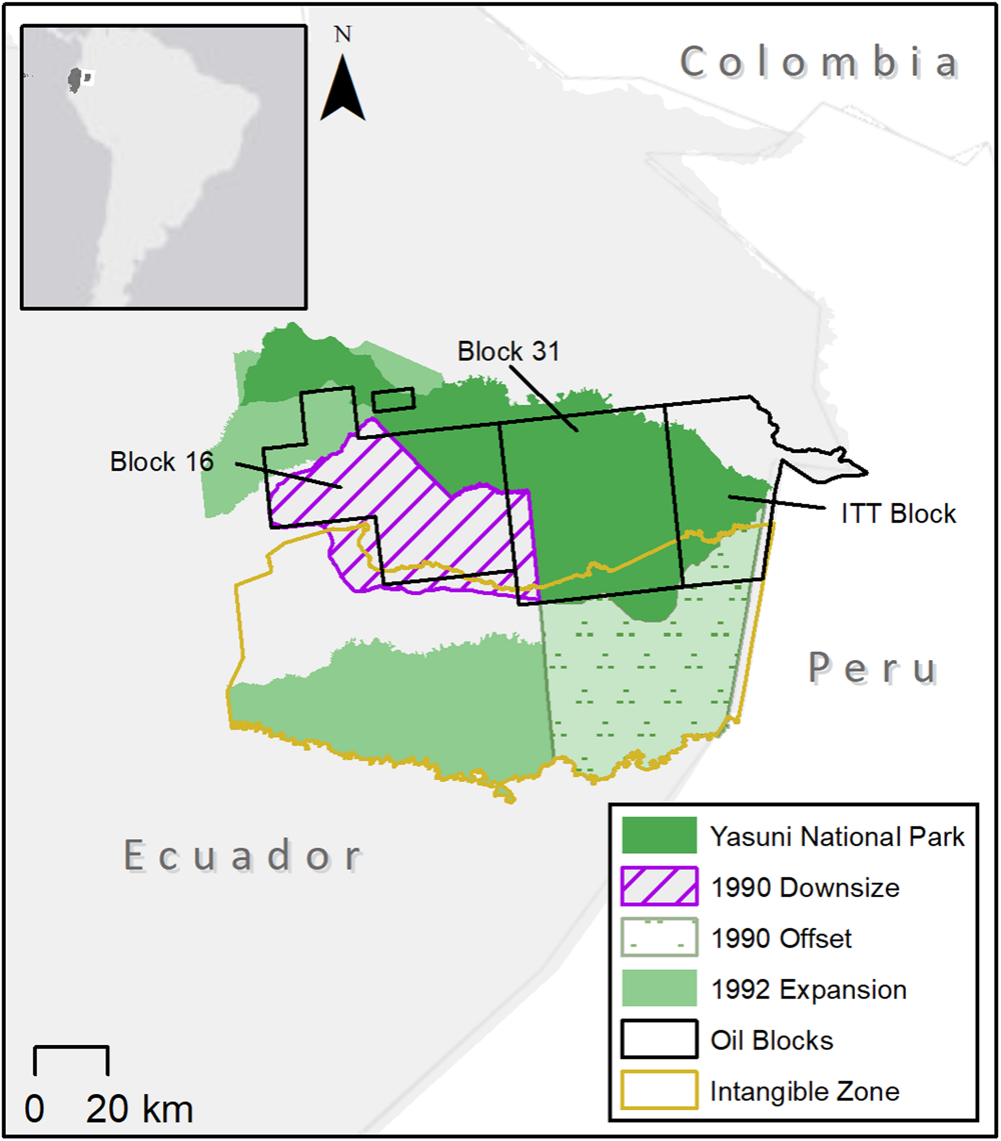
Changes to Yasuní National Park (Ecuador) boundaries and legal restrictions from 1990 to 2018. The park was downgraded in 1992, 1993, 1995, 1997, 2005, and 2006 to authorize oil‐development‐related infrastructure in blocks 16 and 31 and in 2013 to authorize oil drilling. The most recent downgrade in 2013 affected 1% of the park area in the ITT block. A later amendment reduced the area of drilling to 0.1% of the park's area (around 10 km^2^). In 1990, 2,088 km^2^ were removed and 2,460 km^2^ were added as offset. In 1992, 3,177 km^2^ were added to the park. Downgradings in 1995 and 1997 were reversed in 2007 and 2000, respectively. From an original size of 6,331 km^2^, Yasuní National Park now covers 9,820 km^2^ (area figures are not reported in any legal documents for Yasuní; values reported are from spatial data).

The park has a dynamic governance history with numerous downsize and downgrade events (some of which have been offset or reversed) and additions that have resulted in an overall net increase in park area from 6,330 km^2^ to nearly 10,000 km^2^ of Amazonian rainforest (Bass et al. 2010) (Fig. 3). Changes to the protection of the park have been shaped by the interplay between demands for oil and for ecological and cultural conservation (Finer et al. 2008). In 1990, the Ecuadorian government downsized the park by approximately 2,088 km^2^ (and simultaneously expanded protections for 2,460 km^2^) to grant land rights to the Waorani indigenous group (Fig. 3 & Supporting Information). However, land titles associated with the downsized area, which overlaps an oil concession, placed restrictions on indigenous peoples’ rights to prevent oil exploitation (Espinosa 2013). In 1992, the government added approximately 3,177 km^2^ to the southwestern section of the park, expanding protections for fresh water, wetlands, and endemic and native wildlife. Another 6 downgrade events (Supporting Information) authorized oil development‐related infrastructure (e.g., seismic lines, exploration platforms, and heliports) within oil concession blocks 16 and 31, and drilling in the Ishpingo–Tambococha–Tiputini (ITT) oil block (Fig. 3). Partly in response to pressure from the international community (Espinosa 2013), the park was partially upgraded to prohibit industrial‐scale resource extraction through the establishment of the Tagaeri‐Taromenane Intangible Zone (Fig. 3) (Government of Ecuador 1999). In 2007, the government proposed the Yasuní‐ITT initiative to forgo drilling in block 31 and the ITT oil blocks (Espinosa 2013). However, in 2013 and 2014, these upgrades were partly reversed when President Correa canceled the Yasuní ITT Initiative, hence authorizing the expansion of oil exploration on the eastern side of Yasuní (Fig. 3) (Keyman 2015; Vidal 2016).

Negative environmental impacts have been observed within and adjacent to areas downgraded or downsized for oil extraction and related infrastructure, including contamination from oil and wastewater spills (Finer et al. 2008), deforestation, and fragmentation associated with roads and new human settlements (Finer et al. 2015), and unsustainable harvest of wildlife (Suarez et al. 2009). The environmental impacts are likely to extend beyond the extraction area (Finer et al. 2008, 2015; Suarez et al. 2009). Social impacts include the fragmentation of traditional indigenous territories, health problems, and societal destabilization (Swing et al. 2012). As of 2018, deforestation from oil and new settlements in Yasuní was approximately 4.17 km^2^, which exceeds the deforestation limit of 3 km^2^ agreed on by a 2018 referendum (Thieme et al. 2018).

### Virunga National Park

**Figure 4:**
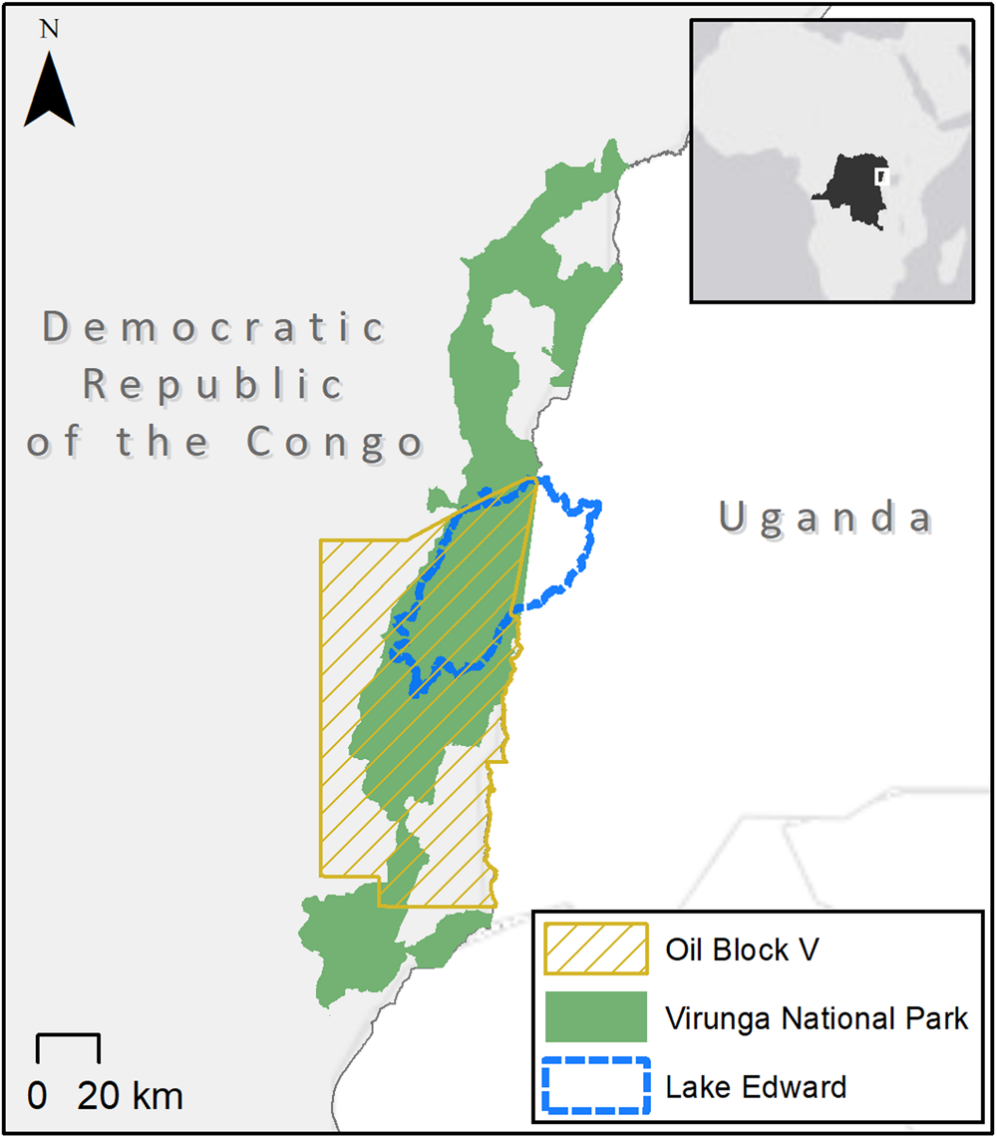
Changes in Virunga National Park (DRC) legal restrictions from 1925 to 2018. Virunga National Park was partially downgraded (2010) for oil exploration in oil block V, which overlaps with 3,897 km^2^ of the park. This downgrade was reversed in 2014 when SOCO International declared it would cease involvement in block V. In 2015 the park was downgraded when the new Hydrocarbon Law made it legal to permit oil exploration in the park and to downsize the park for oil exploitation. In 2018 the government proposed a downsize of 1,720 km^2^ (location unknown) to authorize oil development.

Established in 1925, Virunga National Park is the oldest national park in Africa. Located on the eastern edge of the Democratic Republic of the Congo (DRC) (Fig. 4), Virunga National Park covers over 8,000 km^2^ of diverse ecosystems and geological features (Inogwabini et al. 2005). The park is also known for its megafauna, notably mountain gorillas (*Gorilla beringei*), elephants (*Loxodonta africana*), buffalo (*Syncerus caffer*), and hippopotamuses (*Hippopotamus amphibius*) (UNESCO 2017b). Within the park, 50,000 people directly or indirectly depend on the fishing industry associated with Lake Edward (Dalberg 2013). Virunga National Park became a World Heritage Site in 1979 (UNESCO 1979) and was listed as a Wetland of International Importance under the Ramsar Convention in 1996 (The Ramsar Convention Secretariat 1996).

Since the early 1990s, armed conflicts in and around Virunga National Park have led to poaching and deforestation (UNESCO 1994b). In 2006, the DRC government granted an oil concession for block V (Fig. 4) (SOCO International 2014). A Presidential Decree in 2010 ratified the contract and approved exploration activities, hence downgrading 3,897 km^2^ of the PA that overlapped the oil block (Dalberg 2013), enabling bathymetry survey, a seismic survey, and several geological studies within the park (SOCO International 2014).

In response to opposition from UNESCO and civil society, SOCO halted oil exploration in Virunga in 2014 but advised the DRC government to downsize the park (Gouby 2015). In 2015, the DRC parliament passed the new Hydrocarbon Code (Cabinet of the President of the Republic 2015) enabling oil exploration to be authorized within PAs, which constituted a systemic downgrade of all PAs in the country. The Hydrocarbon Code also made the downsizing and degazettement of PAs legally possible for oil and gas extraction (Cabinet of the President of the Republic 2015). In 2018, the government proposed to downsize 21% (1,720.75 km^2^) of Virunga, as well as 2,767.5 km^2^ of the Salonga National Park, another World Natural Heritage site, to allow oil drilling (Mwarabu & Ross 2018). If enacted, the proposed downsize would likely negatively impact biodiversity and the livelihoods of people who depend on fishing associated with Lake Edward and increase carbon emissions (Peyton 2018).

### Lessons from Cases of PADDD in Iconic Protected Areas

Enacted and proposed PADDD events emerge through a wide range of processes and underlying causes (Mascia & Pallier 2011), and our case studies highlight the complexity and diversity of pressures that lead to PADDD. These cases also offer several insights indicative of the broader phenomenon of PADDD from which one can draw lessons for research and policy.

Most of the PADDD events we described relate to common causes of PADDD (Mascia et al. 2014), including industrial causes, such as infrastructure development (e.g., Yosemite) and resource extraction (e.g., Yasuní, Virunga), and local causes, such as degradation and land grants (e.g., Arabian Oryx Sanctuary, Yasuní). Pressures to develop and exploit natural landscapes are widespread, and even iconic PAs are not immune to them. Yet, little is understood about the conditions under which these pressures translate into PADDD or are resisted. Identifying these patterns is particularly difficult when many PADDD events are not detected and the causes of many events remain unknown (CI & WWF 2019).

Although many elements of the case studies are common to PADDD events more generally, some elements have been overlooked—in particular, the impact of public pressure in moderating or reversing PADDD decisions. For example, pressure from UNESCO and civil society in Virunga National Park led to the reversal of PADDD. The iconic status of these PAs may not be common for all PAs at risk of PADDD, but the cases demonstrate the potential role that greater transparency around PADDD could play in ensuring decisions about the use of protected public lands are perceived as legitimate by the public.

The cases also illustrate the diversity of mechanisms through which PADDD can be enacted (Table 2), such as executive and legislative actions (Yasuní), PA legislation (Yosemite), and other natural resource legislation (e.g., the Hydrocarbon Code in DRC). Some legislative changes can impact all PAs of a particular category (systemic changes), as highlighted in the case of Virunga National Park and previously documented in Peru and Australia (Forrest et al. 2015; Cook et al. 2017). Mechanisms that require agreement from multiple parties to terminate protection may reduce the incidence of PADDD (Hardy et al. 2017).

**Table 2:** History and current status of 4 iconic protected areas and the PADDD^a^ events they have been subjected to

	Yosemite National Park	Yasuní National Park	Arabian Oryx Sanctuary	Virunga National Park
Country	United States	Ecuador	Oman	Democratic Republic of the Congo
Year recognized by UNESCO	1984	1989	1994	1979
Year PADDD enacted (proposed)	1905, 1906, 1892, 1901, 1913	1990, 1992, 1993, 1995, 1997, 2005, 2006, 2013	2007	2010, 2016, 2018
Year PADDD reversed	1964^b^	2000, 2007	NA	2014
Proximate cause	mining & forestry, roads, transmission lines, dam	oil extraction	oil extraction, poaching control	oil extraction
Area gazetted	3,886 km^2^	6,331 km^2^	34,000 km^2^	7,800 km^2^
Area removed	1,445 km^2^	∼2,088 km^2^	∼31,176 km^2^	NA
Legal mechanism to PADDD	legislation	ministerial accords, ministerial resolutions, legislative resolution, executive decree	royal decree	presidential decree, hydrocarbon code
Reprotected + extended	1,116 km^2^	5,577 km^2^	NA	NA
Current size	∼2,995 km^2^	9,820 km^2^	2,824 km^2^	7,800 km^2^
Example impacts	higher level of habitat fragmentation in areas affected by PADDD	oil spills, water contamination, deforestation, fragmentation, social conflicts	limited migration range of Arabian oryx during droughts	threats to biodiversity and local livelihoods
Current status	expanded in 2016 by 1.62 km^2^	oil drilling started on <1% of the park, and may affect larger range	hydrocarbon activities in the downsized areas; remaining area fenced	hydrocarbon law allows PADDD; government considering downsizing for drilling

a Protected area downgrading, downsizing, and degazettement.

b Partial reversal of downsizing that occurred in 1905 and 1906. Fifty‐seven percent of lands downsized were reprotected as wilderness areas.

While providing insights into impacts of individual PADDD events, these case studies highlight the critical gap in our understanding of short and long‐term impacts at national and global scales. Specifically, the Yosemite case study demonstrates that restoring protections may reduce impacts on areas relative to those where PADDD remains in place (Golden Kroner et al. 2016), revealing a temporal element to the impacts of PADDD which is yet to be investigated in other PADDD research.

## PADDD Research Priorities

Drawing on the gaps highlighted by our case studies, and the broader evidence base, we identify several key priorities for future research. First, more regional and country‐level descriptive studies are needed to understand the full extent and history of PADDD. Despite documented PADDD events in 73 countries, systematic PADDD studies are only available for 13 countries (Golden Kroner et al. 2019). These studies reveal that simple comparison of historical versions of PA databases may generate a large number of false positives (i.e., areas that appeared to be removed from protection due to version inconsistencies or boundary corrections instead of true legal changes [Cook et al. 2017; Lewis et al. 2019]), highlighting the value of systematic, in‐country archival research. Future research should also focus on marine PADDD, which remains poorly documented despite a recent wave of PADDD proposals targeting marine protected areas in Australia (Rebgetz 2017; Roberts et al. 2018) and the United States (Milman 2018).

Second, it is critical to gain a better understanding of the risk of PADDD, including contextual factors and underlying drivers that increase or decrease its probability. Differences among national‐level legal contexts likely affect the extent, rates, patterns, and causes of PADDD because the legal context defines what activities are authorized and what changes are legally possible. While pressure from UNESCO has influenced PADDD in iconic PAs, other international laws and treaties, such as the Western Hemisphere Convention (OAS 1940), may further constrain domestic legal processes on PADDD. More research into the legal mechanisms behind PADDD may help reveal the governance arrangements that make PAs more or less vulnerable to PADDD. Likewise, other contextual factors, including land ownership and governance, may play a role in PADDD decisions, which often reflect bargaining between different interests (e.g., Virunga and Yasuní [Tesfaw et al. 2018]). Lastly, the characteristics of PAs, beyond their iconic status, also shape their vulnerability to PADDD. For example, larger PAs ‐ especially those closer to population centers (Symes et al. 2016) ‐ and PAs with higher deforestation rates appear more vulnerable to PADDD (Tesfaw et al. 2018). Further research is required to understand how the legal and other contexts and PA characteristics interact to influence PADDD risks.

The third area for research centers on the social and ecological impacts of PADDD. While preliminary studies suggest a range of impacts of PADDD (e.g., Forrest et al. 2015; Golden Kroner et al. 2016), questions remain. How does PADDD affect surrounding landscapes? What are the impacts on human well‐being? How does impact vary by PADDD process and context? Who benefits from PADDD decisions and who loses? Research addressing historical conflicts and rights of local communities may require the involvement of communities living in areas affected by PADDD to examine livelihood and human rights impacts.

Lastly, further research can improve the understanding of the impermanence of other conservation interventions (e.g., indigenous reserves, privately protected areas), which may count toward the Aichi Target 11 as “other effective area‐based conservation measures” (Secretariat of the CBD 2011). Adapting the PADDD framework to other conservation interventions will broaden understanding of risks, generate a more complete picture of the durability of different interventions, and help in the design of more durable and effective conservation strategies.

## Insights for Conservation Policy

Although further research on PADDD is required to address the drivers of PADDD, available evidence is sufficient to inform initial policy changes to enhance transparency and consistency in PADDD decisions illustrated in our case studies.

One area for policy reform is PADDD tracking and reporting. Currently, no requirements exist at national or international levels to track or report PADDD. International (e.g., CBD) and national policies that mandate standardized PADDD monitoring and reporting and annual public disclosure of enacted and proposed PADDD events would facilitate scientific research, public understanding, and government decision making. For example, such data would allow development and application of key indicators (e.g., number and proximate causes of PADDD events and the number and total area of PAs affected by PADDD) for tracking progress toward targets established by the CBD (e.g., Aichi Targets and their post‐2020 versions).

Another area for reform is the policy processes governing PAs and PADDD. In our case studies PADDD occurred through a variety of mechanisms. These diverse – and often opaque and ill‐defined – procedures create challenges for reporting and tracking and may lead to decisions perceived as illegitimate. Elements of transparent PADDD procedures may include making PADDD proposals public; approving PADDD through the same or higher legal mechanism or instrument, by the same or higher government body, as PA establishment; and requiring comparable levels of scientific assessment and stakeholder consultation as are required to gazette, upsize, or upgrade PAs (Lausche & Burhenne‐Guilmin 2011). For instance, French law follows the principle of parallelism to degazette a PA (i.e., the decision requires the same process as to establish as PA [Guignier & Prieur 2010]).

Vertical and horizontal coordination of institutions and legal frameworks may further reduce ambiguity associated with PADDD decisions. Inconsistent and misaligned policies and development objects often posed threats to the legal protection of PAs, as in the case of Yasuní and Virunga National Parks. When working with governments to create new PAs, international funding institutions could consider reviewing national legal framework to identify and address legal threats to the PA system—possibly similar to the legal and regulatory framework review and reform practiced in the REDD+ readiness phase (UNEP 2005).

The mitigation hierarchy (avoidance, minimization, restoration, and offsetting) can serve as a framework for PADDD governance (ten Kate & Crowe 2014). To avoid PADDD, decision makers could prohibit PADDD in certain types of PAs (e.g., World Heritage Sites) or for certain proximate causes (e.g., fossil fuel extraction) (IUCN 2016). When PADDD decisions are unavoidable, relevant parties should consider limiting the affected area, adopting low‐impact technology, and monitoring approved activities and impacts. Reprotecting downsized or degazetted areas may prevent further damage (Golden Kroner et al. 2016) and may represent priorities for environmental restoration. Finally, PADDD may be offset by upgrading, expanding, or creating PAs with comparable conservation value (e.g., Yosemite and Yasuní) (Lausche & Burhenne‐Guilmin 2011; Pringle 2017), although monitoring and evaluation is necessary to ensure that offsets adequately compensate for losses (Peterson et al. 2018).

## Capacity Needs

Additional human and financial capacity are required to achieve the necessary research advances and policy changes to address PADDD. Providing standard training to local researchers will build the capacity for consistent documentation, reporting, and analysis of PADDD, which enables integrated study and comparative analyses using data collected by different researchers. Such training to local researchers will also expand the expert network for knowledge sharing and collaboration and help identify PADDD research and policy priorities pertinent to the local context. For instance, we established a regional network of PADDD experts in the Amazonian countries, with capacity to identify and document PADDD events through archival research and mapping, by delivering a 5‐day PADDD training and knowledge exchange workshop. Replicating such training in other regions or integrating PADDD training within regional capacity‐building workshops on PA management would help fill data and capacity gaps.

Capacity to host documented PADDD data and to make them publicly accessible can facilitate research and engage civil society to link local PADDD decisions with the global context. Further awareness raising of PADDD within the conservation community and beyond will engage more PADDD data providers and users and contribute to expert capacity to report and review PADDD data via http://PADDDtracker.org and other platforms. Documenting proposed and enacted PADDD events could also shape PADDD decisions, as our case study of the 2014 PADDD in Virunga highlights the role of public engagement to potentially reverse PADDD.

## Conclusion

Widespread legal changes to PAs over the past century (Golden Kroner et al. 2019) have affected even some of the world's most iconic protected lands and waters. With the expansion of PA networks and growing development pressures (Lambin & Meyfroidt 2011; Pouzols et al. 2014; Allan et al. 2017), future conservation success will depend not only on how quickly new areas can be conserved but also on how existing PAs are maintained. Strategic investment in further research, policy reform, and capacity development is key to ensure that PAs can realize their full potential. Collaboration between academics, policymakers, and civil society is essential to achieve the long‐term conservation of nature and sustainable development in a dynamic world.

## Authors’ Contributions

M.B. Mascia and C.M. Rodriguez conceived the idea; S. Qin, R. Golden Kroner, A.T. Tesfaw, C. Cook, and C. Poelking collected data; S. Qin, R. Golden Kroner, C. Cook, R. Braybrook, C.M. Rodriguez, A.T. Tesfaw, and M.B. Mascia wrote the article.

## Supporting information

The full list of enacted or proposed PADDD events identified in the 4 case studies (Appendix S1) is available online. The authors are solely responsible for the content and functionality of these materials. Queries (other than absence of the material) should be directed to the corresponding author.
