# Supplementary material for: Protected area downgrading, downsizing, and degazettement as a threat to iconic protected areas
Source: Conserv Biol. 2019 Jul 17;33(6):1275–85. doi: 10.1111/cobi.13365 (PMC6900045; doi:10.1111/cobi.13365)
Supplement: Supplementary file 1 — The full list of enacted or proposed PADDD events identified in the 4 case studies (Appendix S1) is available online. The authors are solely responsible for the content and functionality of these materials. Queries (other than absence of the material) should be directed to the corresponding author. [file COBI-33-1275-s001.docx]

Appendix S1. Enacted and proposed PADDD events to Yosemite National Park, Arabian Oryx Sanctuary, Yasuní National Park, Virunga National Park, and the Great Barrier Reef Marine Park.

| Protected Area | Event Type | Status | Year PADDD | Proximate  Cause | Area (km^2^)  Affected | Year of Reversal | Legal Document |
| --- | --- | --- | --- | --- | --- | --- | --- |
| Yosemite National Park (USA) | Downgrade | Enacted | 1892 | Infrastructure | unknown | N/A | U.S. Statutes at Large, Vol. 27, Chap. 205, pp. 235-36. "An act granting to the County of Mariposa, in the State of California, the right of way for a free wagon road or turnpike across the Yosemite National Park, in the said state." |
|  | Downgrade | Enacted | 1901 | Infrastructure | unknown | N/A | H.R. 11973 of 1901 |
|  | Downsize | Enacted | 1905 | Forestry | 1,403.8 | N/A | H.R. 173 of 1905 |
|  | Downsize | Enacted | 1906 | Forestry | 41.4 | N/A | H.J.R. 118 of 1906; Public Resolution 27 of 1906 |
|  | Downgrade | Enacted | 1913 | Infrastructure | unknown | N/A | H.R. 7207 of 1913 |
|  | Downgrade | Proposed | 2013 | Forestry | unknown | N/A | H.R. 3188 of 2013 |
| Arabian Oryx Sanctuary (Oman) | Downsize | Enacted | 2007 | Oil and Gas | 31,176 | N/A | Royal Decree 11/2007 |
| Yasuní National Park (Ecuador) | Downsize | Enacted | 1990 | Land Claims | 2,088.0 | N/A | Acuerdo Ministerial No 0191 |
|  | Downgrade | Enacted | 1992 | Oil and Gas | 582.8 | N/A | Resolución INEFAN No 002, 14 Dec 1992 |
|  | Downgrade | Enacted | 1993 | Oil and Gas | 582.8 | N/A | Acuerdo Ministerial 153 |
|  | Downgrade | Enacted | 1995 | Oil and Gas | 1,049.0 | 2007 | Resolución INEFAN No 005, 08 June 1995 |
|  | Downgrade | Enacted | 1997 | Oil and Gas | 582.8 | 2000 | Resolución INEFAN RD No 001, 20 Oct 1997 |
|  | Downgrade | Enacted | 2005 | Oil and Gas | 582.8 | N/A | Resolución Ministerial No 042, 06 July 2005 |
|  | Downgrade | Enacted | 2006 | Oil and Gas | 1,740.0 | N/A | Resolución Ministerial No 099, 28 Nov 2006 |
|  | Downgrade | Enacted | 2013 | Oil and Gas | 9.8 | N/A | Decreto Ejecutivo No 71/2013, Decreto Ejecutivo No 84/2013 |
| Virunga National Park (DRC) | Downgrade | Enacted | 2010 | Oil and Gas | 3,897.0 | 2014 | Ordonnance No. 10/044, 18 June 2010 |
|  | Downgrade | Enacted | 2015 | Oil and Gas | unknown | N/A | Loi n° 15/012 du 1er août 2015 portant régime général des hydrocarbures |
|  | Downsize | Proposed | 2018 | Oil and Gas | 1,720.8 | N/A | unknown |
